# Tenogenic Properties of Mesenchymal Progenitor Cells Are Compromised in an Inflammatory Environment

**DOI:** 10.3390/ijms19092549

**Published:** 2018-08-28

**Authors:** Luisa Brandt, Susanna Schubert, Patrick Scheibe, Walter Brehm, Jan Franzen, Claudia Gross, Janina Burk

**Affiliations:** 1 Saxon Incubator for Clinical Translation (SIKT), University of Leipzig, 04103 Leipzig, Germany; luisa.brandt@sikt.uni-leipzig.de (L.B.); susanna.schubert@sikt.uni-leipzig.de (S.S.); patrick.scheibe@sikt.uni-leipzig.de (P.S.); brehm@vetmed.uni-leipzig.de (W.B.); jan.franzen96@gmx.de (J.F.); claudia_gross1@gmx.de (C.G.); 2 Institute of Veterinary Physiology, University of Leipzig, 04103 Leipzig, Germany; 3 Department for Horses, Veterinary Teaching Hospital, University of Leipzig, 04103 Leipzig, Germany; 4 Institute of Biotechnology, University of Natural Resources and Life Sciences (BOKU), 1190 Vienna, Austria

**Keywords:** mesenchymal stromal cells (MSC), ASC, tendon, extracellular matrix, bioreactor, inflammation, interleukin-1 (IL-1), tumor necrosis factor-α (TNF-α), leukocytes, co-culture

## Abstract

Transplantation of multipotent mesenchymal progenitor cells is a valuable option for treating tendon disease. Tenogenic differentiation leading to cell replacement and subsequent matrix modulation may contribute to the regenerative effects of these cells, but it is unclear whether this occurs in the inflammatory environment of acute tendon disease. Equine adipose-derived stromal cells (ASC) were cultured as monolayers or on decellularized tendon scaffolds in static or dynamic conditions, the latter represented by cyclic stretching. The impact of different inflammatory conditions, as represented by supplementation with interleukin-1β and/or tumor necrosis factor-α or by co-culture with allogeneic peripheral blood leukocytes, on ASC functional properties was investigated. High cytokine concentrations increased ASC proliferation and osteogenic differentiation, but decreased chondrogenic differentiation and ASC viability in scaffold culture, as well as tendon scaffold repopulation, and strongly influenced musculoskeletal gene expression. Effects regarding the latter differed between the monolayer and scaffold cultures. Leukocytes rather decreased ASC proliferation, but had similar effects on viability and musculoskeletal gene expression. This included decreased expression of the tenogenic transcription factor scleraxis by an inflammatory environment throughout culture conditions. The data demonstrate that ASC tenogenic properties are compromised in an inflammatory environment, with relevance to their possible mechanisms of action in acute tendon disease.

## 1. Introduction

Mesenchymal progenitor cells, also referred to as multipotent mesenchymal stromal cells (MSC), are considered as potent therapeutic tools to promote musculoskeletal regeneration. Yet, while advances into the clinical translation of mesenchymal progenitor cell-based therapies are evident, e.g., with respect to cartilage repair [1], progress regarding tendon therapies is slower. The commonly addressed concepts for cell-based tendon therapies include tissue engineering of tendon grafts on the one hand, and direct cell transplantation on the other hand, whereas possible cell-free approaches, such as delivery of extracellular vesicles derived from mesenchymal progenitor cells, are still in early stages [2,3,4]. Direct progenitor cell transplantation to the site of injury is currently the most promising approach to treat conditions involving large load-bearing tendons such as the human Achilles tendon. Interestingly, this concept is already in clinical use in equine veterinary medicine for more than a decade [5], which is of particular interest for human medicine, as the horse represents the only available model animal for naturally occurring large tendon disease [6], and because human and equine mesenchymal progenitor cells have been shown to display a high degree of similarity [7]. Although the underlying regenerative mechanisms are not yet fully understood and clinical evidence is still at a preliminary stage, studies in equine naturally occurring tendon disease have so far demonstrated safety as well as beneficial effects [8,9,10].

The cell transplantation approach overcomes the challenge to engineer tendon constructs that adequately reproduce the exceptional biomechanical properties that would be required for the replacement of large load-bearing tendons [11]. In contrast to in vitro tissue engineering, it involves guided local injections of progenitor cells into the tendon lesions existing during the (sub)acute phases of tendon disease or injury. Thereby, it pursues the concept of in vivo tissue engineering, with the environment within the tendon lesion, including the tendon extracellular matrix and the physiological mechanical load, directing the fate of injected progenitor cells to tenogenic differentiation. At the same time, the remaining healthy tendon tissue can provide structural support. However, by now, it has become clear that mechanisms of action of mesenchymal progenitor cells are far more complex, and the original hypothesis that tenogenic differentiation and tenocyte replacement represent their most important regenerative mechanisms, has repeatedly been questioned. Instead, immuno- and matrix modulation, as well as trophic support for resident cells, are considered to play at least equally important roles [12,13]. 

Investigating the regenerative mechanisms of mesenchymal progenitor cells in tendon healing is crucial to developing evidence-based therapeutic concepts. There is still tremendous uncertainty regarding the fundamental aspects, among others, including the choice of cell source and expansion procedures, cell numbers to be injected, or the optimal timing for the treatment. The latter is likely to be of particular importance, as mechanisms of action of mesenchymal progenitor cells are known to be strongly context-dependent [14,15]. Therefore, as the local environment within the tendon lesion changes during the course of the disease or during the healing process after acute injury [16,17], the time point chosen for cell delivery will impact on the therapeutic efficacy. Investigating the cellular regenerative mechanisms in specific contexts, however, requires in vitro and in vivo disease models that adequately reflect these contexts.

Providing a basis for the long-term regenerative effects of the mesenchymal progenitor cells in tendon healing, there has been evidence of a remarkable long-term persistence and engraftment of cells that had been injected into equine tendon lesions [18,19,20], although this may only be true for a small fraction of the injected cells [21]. Furthermore, tenogenic differentiation could be induced by naturally occurring stimuli such as tendon extracellular matrix and cyclic loading, as demonstrated by our own [22] and others’ [23,24] previous work, in which progenitor cell-seeded decellularized tendon matrices were subjected to dynamic culture in customized cyclic strain bioreactors. These findings demonstrate that the tenogenic differentiation of mesenchymal progenitor cells, potentially followed by matrix-modulating activities [25], could contribute to their regenerative mechanisms in tendon healing. However, the latter studies do not acknowledge that feasible treatment protocols commonly involve the injection of these cells during the inflammatory phase of tendon healing. This implies the presence of leukocytes and pro-inflammatory cytokines such as interleukin-1β (IL-1β) and (tumor necrosis factor-α) TNF-α, which have been shown to impact on tenocytes and foster extracellular matrix degradation [26,27,28,29,30]. While there is also evidence that mesenchymal progenitor cells context-sensitively interact with leukocytes, with their modulatory mechanisms being activated upon inflammatory stimulation [31,32], it is not yet known in which way inflammatory environment impacts on their tenogenic differentiation.

This study aimed to investigate whether inflammatory environment impacts on functional characteristics of adipose-derived equine mesenchymal progenitor/stromal cells (ASC), with a focus on their tenogenic properties in a three-dimensional dynamic in vitro model. The inflammatory environment was reflected by either direct co-culture with non-activated or concanavalin A-activated leukocytes, or by the addition of pro-inflammatory cytokines, the latter being used at low concentrations similar to those found in vivo, as well as at high concentrations as those commonly applied in vitro. We found that not only high cytokine concentrations, but also the presence of leukocytes impacted on ASC functional characteristics and compromised tenogenic properties.

## 2. Results

### 2.1. Cell Morphology, Proliferation, and Trilineage Differentiation

**Figure 1:**
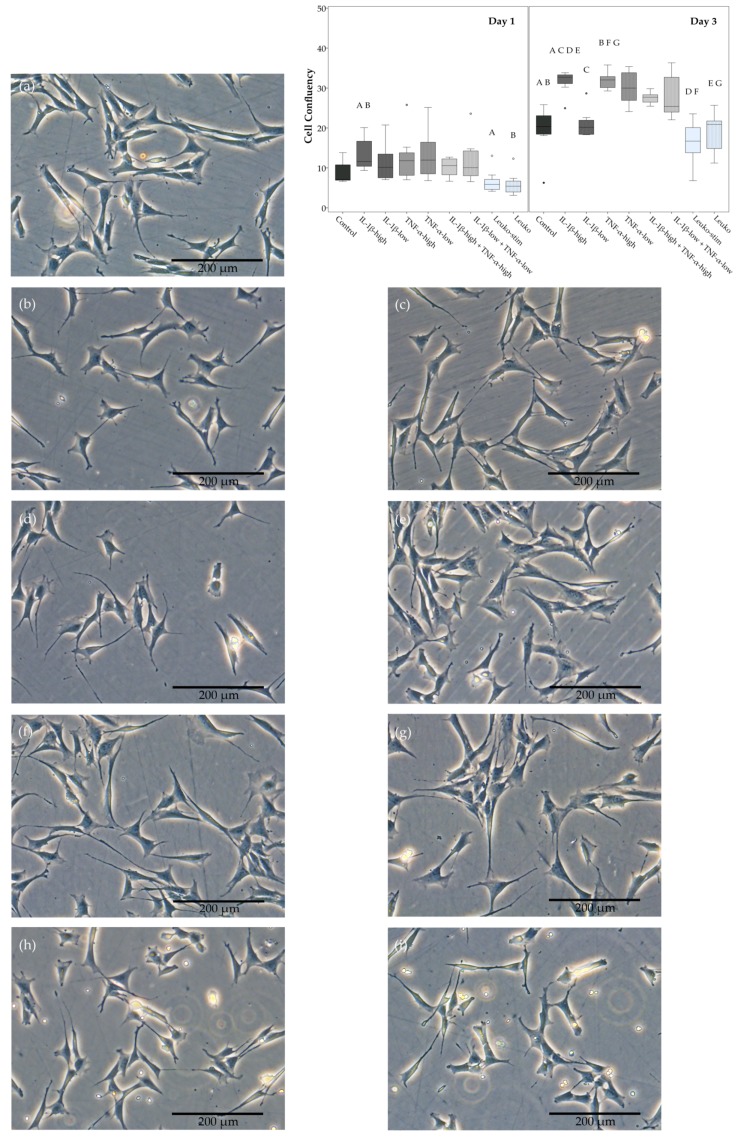
Adipose-derived stromal cell (ASC) morphology and proliferation. Exemplary phase contrast images of ASC monolayer cultures in different inflammatory environments ((**a**) control; (**b**) IL-1β-high (10 ng/mL); (**c**) IL-1β-low (0.01 ng/mL); (**d**) TNF-α-high (50 ng/mL); (**e**) TNF-α-low (0.1 ng/mL); (**f**) IL-1β-high + TNF-α-high; (**g**) IL-1β-low + TNF-α-low; (**h**) co-culture with activated leukocytes (Leuko-stim); (**i**) co-culture with non-activated leukocytes (Leuko)) obtained at Day 1, and boxplot displaying confluency as a measurement of ASC proliferation. Data were obtained by quantitative analysis of phase contrast images from Day 1 and Day 3. Groups displayed by boxes sharing the same letter are significantly different from each other (*p* < 0.05; *n* = 7).

ASC showed a spindle-shaped fibroblast-like appearance without inflammatory stimulation. In inflammatory environments, their cell bodies became flatter with further cell processes, regardless of the cytokine concentration or the leukocyte activation (Figure 1).

ASC proliferation in monolayer culture was enhanced in the presence of the pro-inflammatory cytokines IL-1β and TNF-α, alone as well as when combined. The confluency of the monolayers at Day 3 was significantly higher at high concentrations of IL-1β, as well as TNF-α. A similar trend was observed for the corresponding low cytokine concentrations, except for IL-1β-low, and for the combined cytokines. Co-culture with activated as well as non-activated leukocytes, however, rather led to lower ASC confluency. The generation times as calculated from exemplary samples confirmed that confluency was representative for cell proliferation (Table S1). Details regarding the significance of differences between groups are shown in Figure 1. 

**Figure 2:**
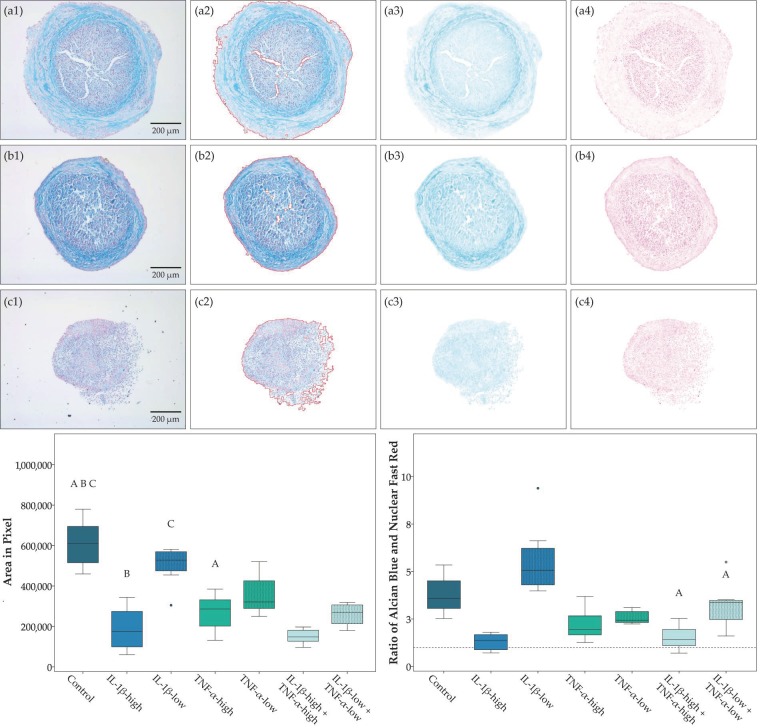
Chondrogenic differentiation. Exemplary images of micromasses obtained after chondrogenic differentiation in different inflammatory environments (first row, **a1** to **a4**: control; second row, **b1** to **b4**: IL-1β-high (10 ng/mL) + TNF-α-high (50 ng/mL); third row, **c1** to **c4**: IL-1β-low (0.01 ng/mL) + TNF-α-low (0.1 ng/mL)) and Alcian blue and Nuclear Fast Red staining; the first column displays the original images (**a1** to **c1**); the second column shows the brightness-corrected and masked areas, where the mask boundary is indicated by the red line (**a2** to **c2**), the next two columns show a colorized representation of the staining concentration images from the color deconvolution for Alcian blue (**a3** to **c3**) and Nuclear Fast Red (**a4** to **c4**). Boxplots display the pellet area as a measurement for micromass size and the ratio of Alcian blue to Nuclear Fast Red staining as a measurement for cartilaginous matrix deposition. Groups displayed by boxes sharing the same letter are significantly different from each other (*p* < 0.05; *n* = 7).

**Figure 3:**
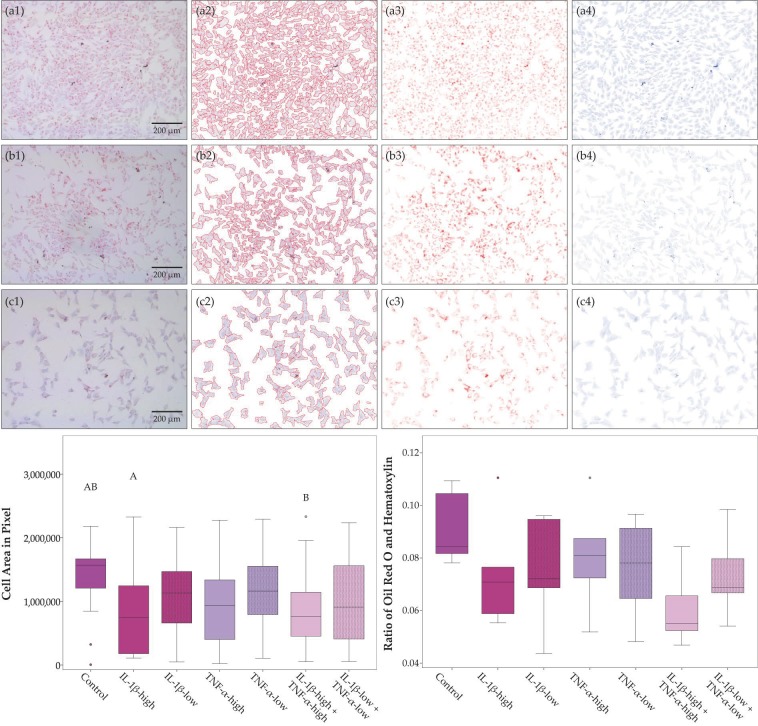
Adipogenic differentiation. Exemplary images of adipose-derived stromal cells (ASC) after adipogenic differentiation in different inflammatory environments (first row, **a1** to **a4**: control; second row, **b1** to **b4**: IL-1β-high (10 ng/mL) + TNF-α-high (50 ng/mL); third row, **c1** to **c4**: IL-1β-low (0.01 ng/mL) + TNF-α-low (0.1 ng/mL)) and Oil Red O and hematoxylin staining; the first column displays the original images (**a1** to **c1**); the second column shows the brightness-corrected and masked areas, where the mask boundary is indicated by the red line (**a2** to **c2**), the next two columns show a colorized representation of the staining concentration images from the color deconvolution for Oil Red O (**a3** to **c3**) and hematoxylin (a4 to c4). The boxplots display the cell area as a measurement for cell proliferation and the ratio of Oil Red O to hematoxylin staining as a measurement for accumulation of intracellular lipid vacuoles. Groups displayed by boxes sharing the same letter are significantly different from each other (*p* < 0.05; *n* = 4).

**Figure 4:**
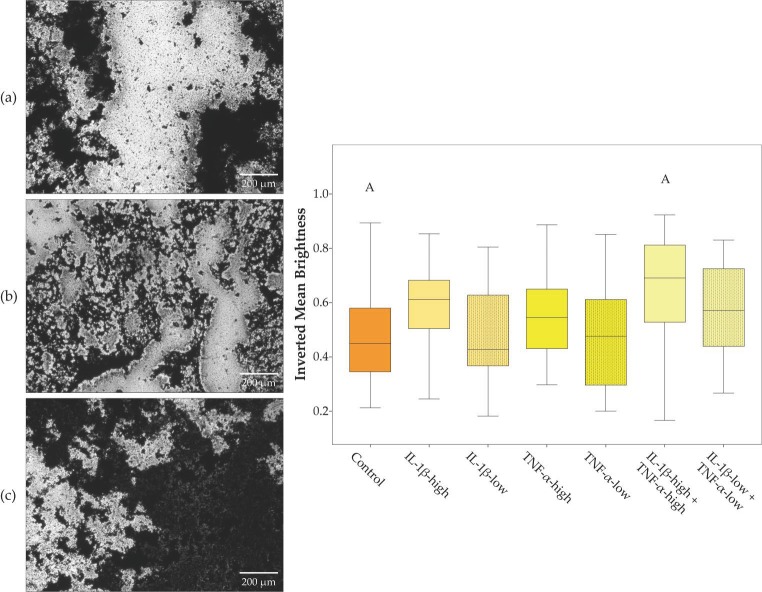
Osteogenic differentiation. Exemplary images of adipose-derived stromal cells (ASC) after osteogenic differentiation in different inflammatory environments (**a**: control; **b**: IL-1β-high (10 ng/mL) + TNF-α-high (50 ng/mL); **c**: IL-1β-low (0.01 ng/mL) + TNF-α-low (0.1 ng/mL)) and von Kossa staining; the boxplot displays the inverted mean brightness of images as a measurement for deposition of mineralized matrix. Groups displayed by boxes sharing the same letter are significantly different from each other (*p* < 0.05; *n* = 7).

Chondrogenic differentiation in pellet culture was compromised in the presence of pro-inflammatory cytokines, except for IL-1β-low. The obtained micromasses were smaller in all groups with high cytokine concentrations (IL-1β, TNF-α and both cytokines combined), and the proportion of the micromasses stained by Alcian blue was lowest in presence of IL-1β-high, alone or combined, indicating reduced deposition of cartilaginous matrix (Figure 2). Moreover, no micromasses were obtained at all from two donor animals in the presence of IL-1β-high and TNF-α-high and from three donor animals in the presence of both cytokines combined. Adipogenic differentiation led to corresponding results; however, differences did not reach significance (Figure 3). In contrast, osteogenic differentiation was promoted in the presence of pro-inflammatory cytokines. This was significant when IL-1β and TNF-α were combined at high concentrations, based on the lower brightness of images obtained from the respective von Kossa-stained samples, indicating a stronger deposition of mineralized matrix (Figure 4).

### 2.2. Cell Viability 

The impact of inflammatory environment on ASC viability was investigated in different culture conditions (monolayer culture and static culture on tendon extracellular matrix scaffolds and dynamic culture on tendon extracellular matrix scaffolds in a cyclic strain bioreactor). As indicated by quantitative analysis of dead cells in LIVE/DEAD™-stained samples, regarding the control cultures without inflammatory stimulation, there were no significant differences between Day 1 and Day 3 or between static and dynamic scaffold cultures.

**Figure 5:**
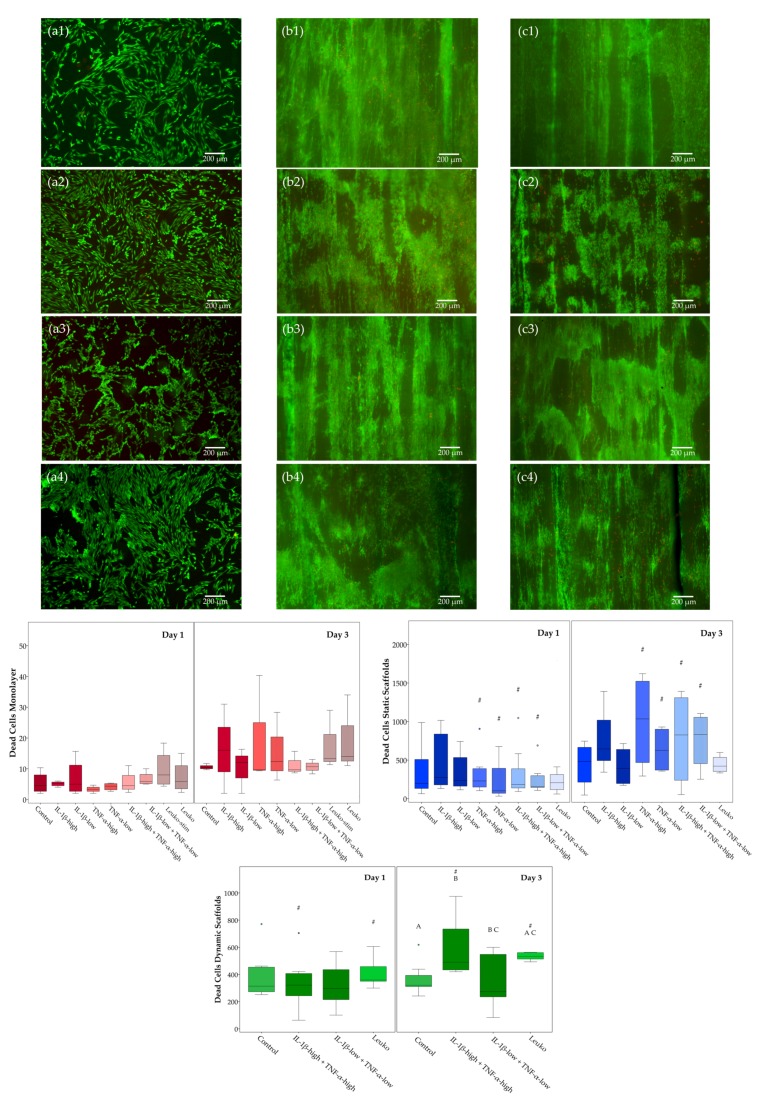
LIFE/DEAD™ staining and analysis of dead cells. Exemplary images of adipose-derived stromal cells (ASC) in monolayer culture (first column, **a1** to **a4**), static scaffold culture (second column, **b1** to **b4**), and dynamic scaffold culture (third column, **c1** to **c4**) in different inflammatory environments (first row, **a1** to **c1**: control; second row, **a2** to **c2**: IL-1β-high (10 ng/mL) + TNF-α-high (50 ng/mL); third row, **a3** to **c3**: IL-1β-low (0.01 ng/mL) + TNF-α-low (0.1 ng/mL); fourth row, **a4** to **c4**: co-culture with non-activated leukocytes (Leuko)) after LIVE/DEAD™ staining at day 3; viable cells are shown in green, dead cells in red; boxplots display the numbers of dead cells in the different culture conditions. Groups displayed by boxes sharing the same letter are significantly different from each other; hash marks (#) indicate significant differences between Day 1 and Day 3 (*p* < 0.05; *n* = 7).

The effects of inflammatory stimulation were more pronounced in scaffold cultures. The highest numbers of dead cells were found in static scaffold cultures with inflammatory stimulation by cytokines at Day 3, but variability was also high and differences were not significant compared to the control. However, in all groups including TNF-α, there was a significant increase in dead cell numbers from Day 1 to Day 3, whereas there was no effect of co-culture with leukocytes. In dynamic scaffold cultures, although numbers of dead cells remained overall lower, and the effects of the inflammatory environment were consistently observed, IL-1β combined with TNF-α at high concentrations as well as co-culture with non-activated leukocytes leading to significantly increased numbers of dead cells at Day 3. In contrast, in monolayer cultures, no significant effects were observed at all, including that no differences were found between activated and non-activated leukocytes (Figure 5).

### 2.3. Musculoskeletal Marker Expression

The impact of inflammatory environment on musculoskeletal gene expression was again investigated in different culture conditions (monolayer culture and static culture on tendon extracellular matrix scaffolds, and dynamic culture on tendon extracellular matrix scaffolds in a cyclic strain bioreactor). Relative target gene expression ratios in the respective control groups, reflecting effects of these different culture conditions without inflammatory stimulation, are shown in Figure S1. Briefly, (dynamic) scaffold culture increased the expression of collagen 3A1, decorin and osteopontin and decreased expression of mohawk and smad8, compared to the monolayer culture. For details regarding the significance of differences between groups see Figure S1. The expression patterns of collagen 1A2, scleraxis, and tenascin-C, which have previously been described to discriminate healthy tendon tissue, corresponded better to the pattern seen in tendon tissue [33], in the (dynamic) scaffold cultures than in the monolayer culture, with high collagen 1A2 expression, medium scleraxis expression, and low but still evident tenascin-C expression. However, the collagen 1 to collagen 3 expression ratio, which was also used as a marker for healthy tendon tissue, tended to be decreased in (dynamic) scaffold culture, possibly due to the native collagen 1 present in the scaffold (Figure S2). The following subsections focus on the effects of the different inflammatory environments investigated.

#### 2.3.1. Monolayer Cultures 

**Figure 6:**
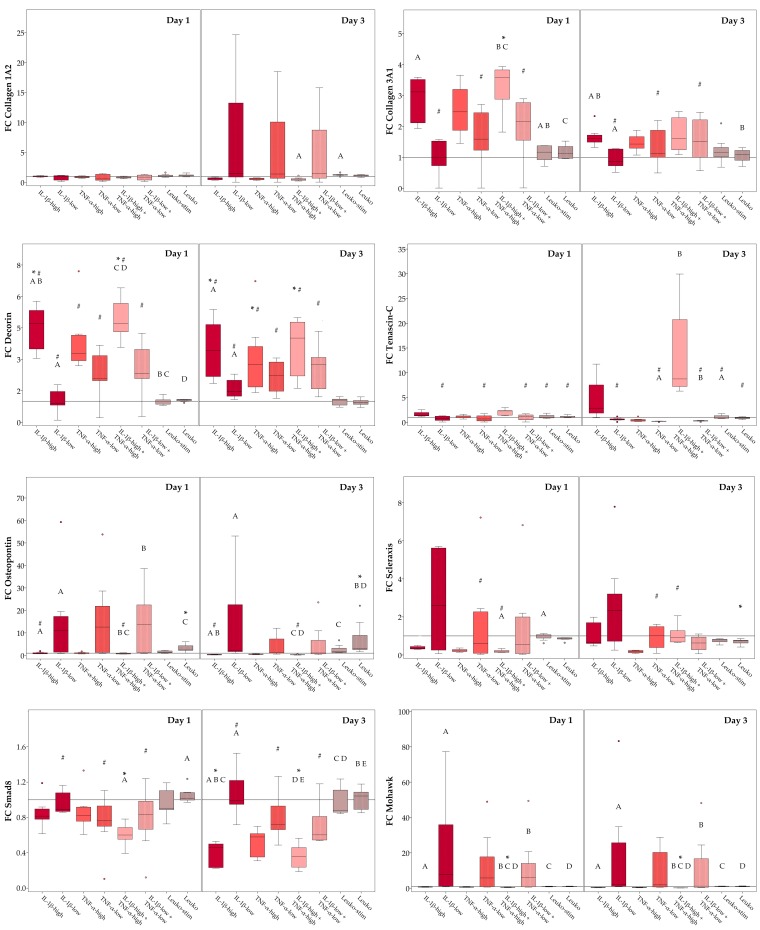
Musculoskeletal marker expression in monolayer culture. Boxplots displaying relative gene expression of monolayer adipose-derived stromal cells (ASC) in inflammatory environments (IL-1β-high: 10 ng/mL; IL-1β-low: 0.01 ng/mL; TNF-α-high: 50 ng/mL; TNF-α-low: 0.1 ng/mL; Leuko-stim: co-culture with activated leukocytes; Leuko: co-culture with non-activated leukocytes), presented as fold-change (FC) compared to the respective control (indicated by the line at 1). Stars indicate significant differences compared to the control; groups displayed by boxes sharing the same letter are significantly different from each other; hash marks indicate significant differences between Day 1 and Day 3 (*p* < 0.05; *n* = 7).

First, we compared all different inflammatory conditions tested regarding their influence on gene expression of musculoskeletal and tendon markers in monolayer ASC cultures. As expected, high cytokine concentrations had the most substantial influence on gene expression, decreasing expression of the extracellular matrix molecules collagen 1A2 and osteopontin, as well as the intracellular molecules scleraxis, mohawk and smad8 and increasing expression of the extracellular matrix molecules collagen 3A1 and decorin. For these target genes, gene expression in the presence of IL-1β, TNF-α or the combination of both followed an overall similar trend. Only tenascin-C tended to be upregulated in the presence of IL-1β, but downregulated in the presence of TNF-α. In contrast, low cytokine concentrations rather led to a strong variation of gene expression levels within groups, although with some differences between gene expression levels at Day 1 and Day 3, indicating that ASC from different donors adapt to these (patho)physiological cytokine concentrations in different ways. Co-culture with leukocytes did not have a significant impact on the expression of most target genes, yet it increased osteopontin and decreased scleraxis expression, indicating that leukocytes may counteract tenogenic differentiation. Interestingly, there was never a significant difference between gene expression levels in co-cultures with activated leukocytes compared to non-activated leukocytes; thus, co-cultures with activated leukocytes were omitted in the following scaffold culture experiments. Fold-change gene expression levels, as well as the details regarding the significance of differences between groups, are shown in Figure 6.

#### 2.3.2. Static Scaffold Cultures

**Figure 7:**
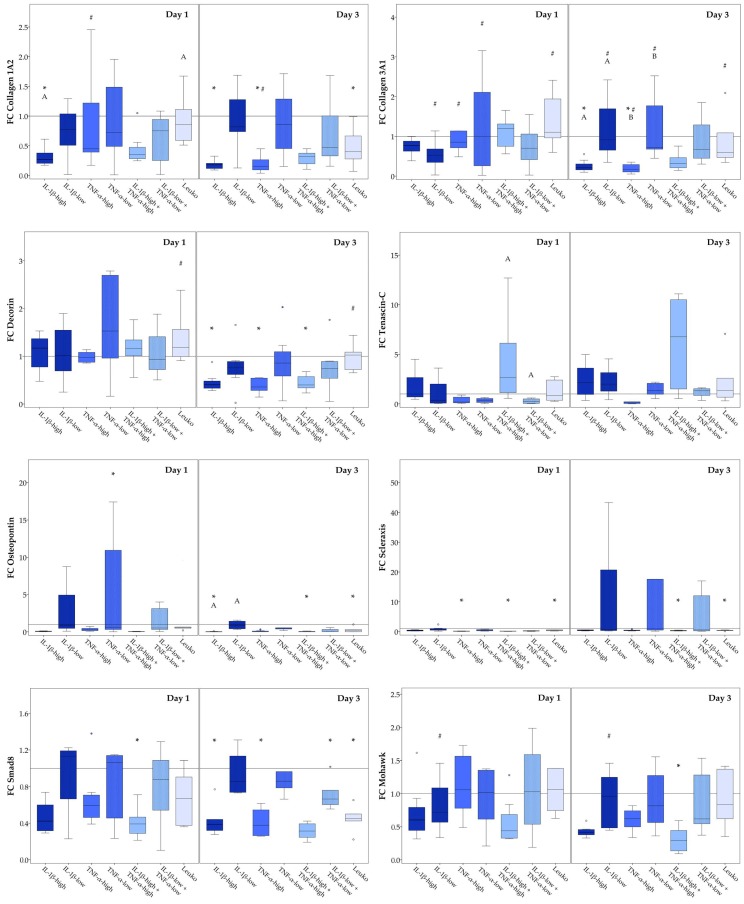
Musculoskeletal marker expression in static scaffold culture. Boxplots displaying the relative gene expression of static scaffold culture adipose-derived stromal cells (ASC) in inflammatory environments (IL-1β-high: 10 ng/mL; IL-1β-low: 0.01 ng/mL; TNF-α-high: 50 ng/mL; TNF-α-low: 0.1 ng/mL; Leuko: co-culture with non-activated leukocytes), presented as fold change (FC) to the respective control (indicated by the line at 1). Stars (*) indicate significant differences compared to the control; groups displayed by boxes sharing the same letter are significantly different from each other; hash marks (#) indicate significant differences between Day 1 and Day 3 (*p* < 0.05; *n* = 7).

Next, we compared the different inflammatory conditions, except for the co-culture with activated leukocytes, regarding their influence on musculoskeletal marker expression in static scaffold cultures. Again, high concentrations of cytokines decreased the expression of the extracellular matrix molecules collagen 1A2 and osteopontin, as well as expression of the intracellular molecules scleraxis, mohawk, and smad8. Furthermore, the different cytokines again widely led to the same effects except when considering tenascin-C expression. However, in contrast to the monolayer cultures, collagen 3A1 and decorin expression levels were also decreased in the presence of high cytokine concentrations. Similar to that observed in the monolayer cultures, low cytokine concentrations led to varying gene expression levels between donors, with no major effects but with some differences in gene expression in these groups between Days 1 and 3. Interestingly, in scaffold culture, the presence of non-activated leukocytes affected the expression of more target genes than in monolayer culture, including downregulation of collagen 1A2, scleraxis, and smad8, as well as osteopontin, the latter standing in contrast to its upregulation in the presence of leukocytes in monolayer culture. Furthermore, leukocytes induced a decrease in collagen 3A1 and decorin expression levels between day 1 and 3. Fold-change gene expression levels, as well as the details regarding the significance of differences between groups, are shown in Figure 7.

#### 2.3.3. Dynamic Scaffold Cultures

**Figure 8:**
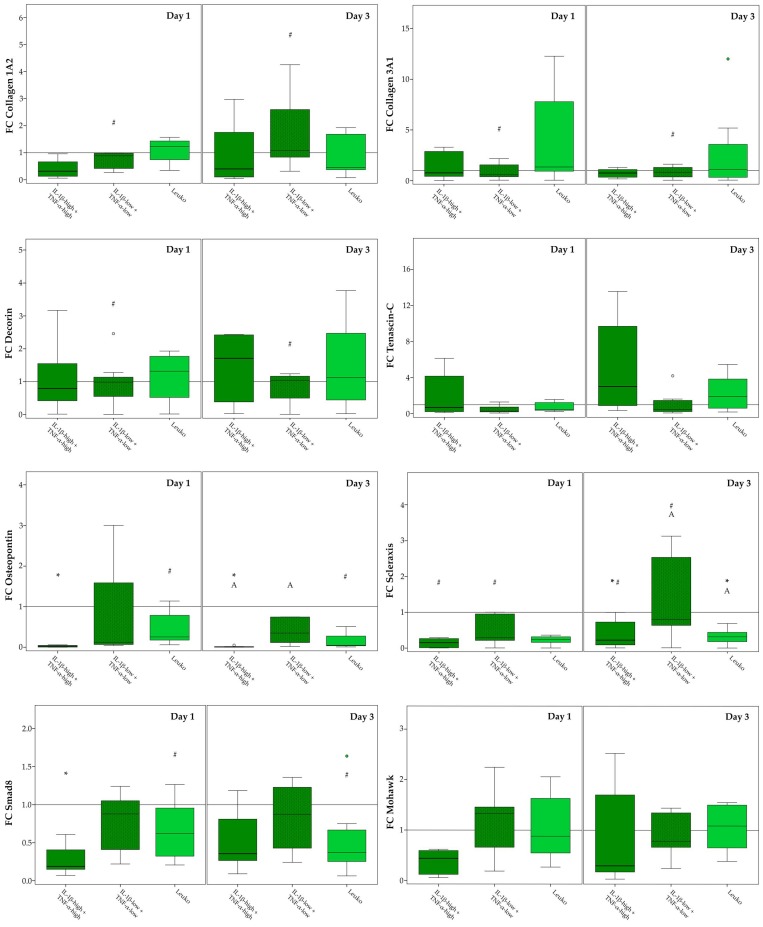
Musculoskeletal marker expression in dynamic scaffold culture. Boxplots displaying the relative gene expression of dynamic scaffold culture adipose-derived stromal cells (ASC) in inflammatory environments (IL-1β-high: 10 ng/mL; IL-1β-low: 0.01 ng/mL; TNF-α-high: 50 ng/mL; TNF-α-low: 0.1 ng/mL; Leuko: co-culture with non-activated leukocytes), presented as fold change (FC) to the respective control (indicated by the line at 1). Stars indicate significant differences as compared to the control; groups displayed by boxes sharing the same letter are significantly different from each other; hash marks indicate significant differences between Day 1 and Day 3 (*p* < 0.05; *n* = 7).

Finally, we tested selected inflammatory conditions, including combined IL-1β and TNF-α at high and low concentrations, as well as co-culture with non-activated leukocytes, in dynamic scaffold cultures. Interestingly, in this dynamic environment that more closely mimicked in vivo conditions, inflammatory conditions appeared to have less impact on the overall musculoskeletal gene expression. Yet, high cytokine concentrations still decreased the expression levels of osteopontin, scleraxis, and smad8, and co-culture with non-activated leukocytes still decreased scleraxis expression (Figure 8). As a consequence, the tendon-like expression pattern of collagen 1A2, scleraxis, and tenascin-C observed without inflammatory stimulation was altered by high cytokine concentrations as well as leukocytes. High cytokine concentrations also tended to further decrease the collagen 1 to collagen 3 expression ratio, but not significantly (Figure S2). Differences in gene expression between day 1 and day 3 were most often found in the presence of low cytokine concentrations, corresponding to the results in monolayers and static scaffold cultures. Fold change gene expression levels, as well as the details regarding the significance of differences between groups, are shown in Figure 8.

### 2.4. ASC Alignment on Tendon Scaffolds and Scaffold Repopulation

As demonstrated qualitatively by LIVE/DEAD™ staining, there were high numbers of viable ASC that showed an alignment within the tendon scaffold in contrast to their more random and swarm-like appearance in monolayer culture. However, in dynamic scaffold cultures and particularly in inflammatory environment, there were also cell aggregates that accumulated on the scaffold surface in a characteristic pattern, potentially due to the combination of shear forces and leukocyte-ASC interactions (Figure 5). 

**Figure 9:**
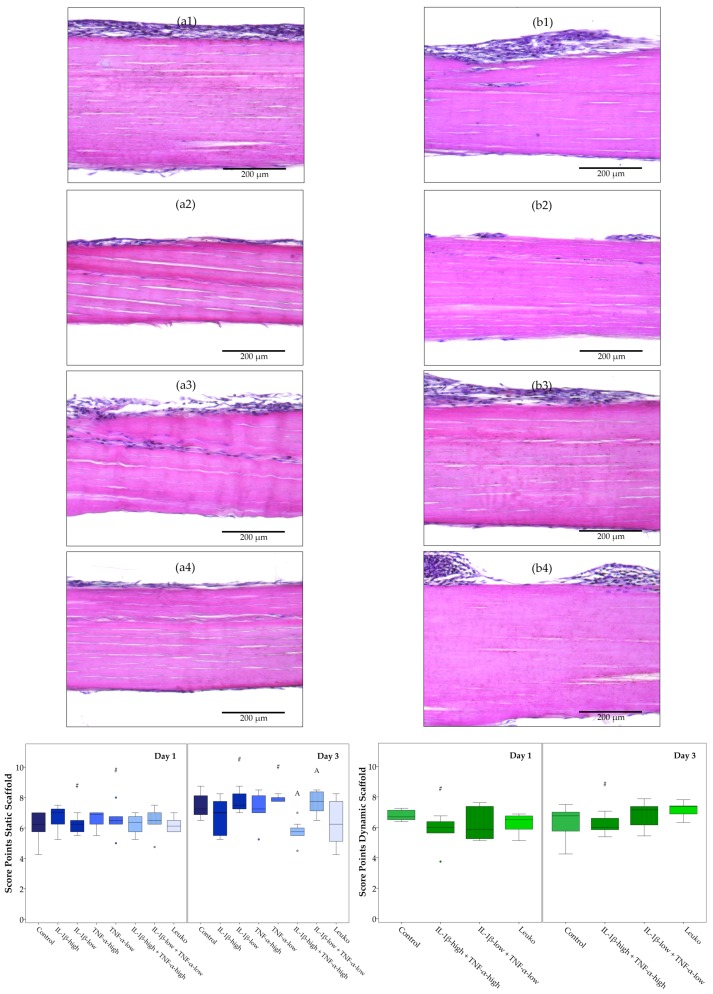
Histological evaluation of adipose-derived stromal cell (ASC)-seeded tendon scaffolds. Exemplary images of hematoxylin and eosin-stained sections of ASC-seeded tendon scaffolds at Day 3, with the seeded scaffold surface displayed at the top (first column, **a1** to **a4**: static scaffold culture; second column, **b1** to **b4**: dynamic scaffold culture) in different inflammatory environments (first row, **a1** and **b1**: control; second row, **a2** and **b2**: IL-1β-high (10 ng/mL) + TNF-α-high (50 ng/mL); third row, **a3** and **b3**: IL-1β-low (0.01 ng/mL) + TNF-α-low (0.1 ng/mL); fourth row, **a4** and **b4**: co-culture with non-activated leukocytes (Leuko)), and boxplots displaying the total score points for the evaluation of scaffold repopulation efficiency. Groups displayed by boxes sharing the same letter are significantly different from each other; hash marks (#) indicate significant differences between Day 1 and Day 3 (*p* < 0.05; *n* = 7).

Histologic evaluation showed that ASCs did not remain restricted to populating the seeded scaffold surface, but they also attached to the opposite scaffold surface and partially integrated between the tendon fibers, which was overall more evident at Day 3. Pro-inflammatory cytokines tended to reduce the efficiency of tendon scaffold repopulation. However, co-culture with leukocytes did not alter scaffold repopulation. The cell distribution at the seeded scaffold surface was only reduced in dynamic scaffold cultures in the presence of cytokines at high concentrations at Day 1. However, cell attachment on the opposite scaffold surface was compromised in the presence of combined cytokines in static and dynamic scaffold cultures on Day 3. Cell integration tended to be lower in dynamic scaffold cultures with inflammatory stimulation, which was significant comparing static and dynamic scaffold cultures incubated with combined cytokines at low concentrations at Days 1 and 3. Only IL-1β-low used in static scaffold cultures appeared to promote cell integration between Day 1 and Day 3. Regarding the overall histology score, however, fewer significant differences were found, but the scores were reduced in the presence of combined cytokines at high concentrations in static scaffold cultures at Day 3, and in dynamic scaffold cultures at Day 1 (Figure 9 and Figure S3).

## 3. Discussion

The current study is the first to provide evidence that the tenogenic capacities of mesenchymal progenitor cells are compromised not only in artificial inflammatory environment represented by high concentrations of pro-inflammatory cytokines, but also in the presence of leukocytes. Overall, while inflammatory environment increased ASC proliferation and osteogenic differentiation, it decreased cell viability, chondrogenic and adipogenic differentiation potential, the efficiency of tendon scaffold repopulation, and the expression of musculoskeletal markers, including the tenogenic transcription factor scleraxis. These findings add to the knowledge on ASC mechanisms of action and have direct implications for cell transplantation therapy in acute tendon disease.

The in vitro models used in the current study are unique in that we aimed to overcome the highly artificial character of standard in vitro culture models in a step-wise manner. This implied that on the one hand, cell culture techniques proceeded from standard monolayer culture to static, and finally to dynamic culture on native extracellular tendon matrix scaffolds, applying moderate cyclic strain reflecting rehabilitation exercises [22]. On the other hand, we chose different inflammatory stimuli, for which we used the pro-inflammatory cytokines IL-1β and TNF-α to facilitate standardization and comparisons to other studies, as well as direct co-culture with non-activated or concanavalin A-activated leukocytes to mimic a pathophysiological in vivo environment more closely. The effects of these inflammatory stimulation regimes were observed over a period of three days. This maintained feasibility of the co-culture experiments with leukocytes, including their short-lived granulocyte subpopulation, and corresponds to the relatively short acute inflammatory phase in tendon healing [34]. 

The dynamic scaffold culture maintained a tendon-like gene expression pattern in the ASC, with high levels of collagen 1A2, medium levels of scleraxis and low levels of tenascin-C expression. This pattern had previously been found to discriminate healthy tendon from other musculoskeletal tissues as well as monolayer-cultured cells [33]. Furthermore, in accordance with previous data [22], dynamic scaffold culture led to an increased collagen 3A1 and decorin expression. This corresponds to findings in tendon disease [17,35], thus indicating that the model reflects tendon repair. However, the increase in scleraxis expression induced by scaffold culture and cyclic stretching, which was observed in previous studies [22,23], could not be reproduced in the current study. This may be due to small differences between study protocols, or due to differences between the biological materials used, and it underlines the challenges encountered with increasing complexity of in vitro models. In the current context, relying on materials from different donors not only with regard to cell recovery but also for scaffold production, it appears particularly important to rigorously standardize scaffold production by characterizing the scaffolds obtained from different donors.

The effects of the different inflammatory stimuli were widely similar, but by far not the same across culture conditions, with a few important differences. The increase in dead cell numbers upon inflammatory stimulation was only pronounced in scaffold cultures. Furthermore, downregulation of collagen 3A1 and decorin by pro-inflammatory cytokines was observed in static scaffold culture, whereas these genes were upregulated in monolayer culture. Similarly, osteopontin expression was increased in the presence of leukocytes in monolayer culture but decreased in static scaffold culture. In addition, co-culture with leukocytes affected the expression of more target genes in static scaffold cultures than in the other culture conditions. Finally, more pronounced effects of pro-inflammatory cytokines on scaffold repopulation were seen in the dynamic scaffold cultures. Seeing such differences between the effects of inflammatory stimulation in different cell culture models was not unexpected. In a previous study, we found that the tenogenic effects of transforming growth factor (TGF)-β3 seen in monolayer culture are strongly altered by tendon scaffold culture [36]. Furthermore, it has been shown that immunomodulatory properties of mesenchymal progenitor cells are altered by a three-dimensional scaffold culture environment compared to two-dimensional culture [37]. It has also been shown that cells are more sensitive regarding the cytotoxicity of different substances when cultured in three-dimensional conditions, demonstrating that drug testing in a standard monolayer cultures alone is not predictive enough for in vivo toxicity [38]. In accordance with this, our study demonstrates the importance of the in vitro model design, and although there are still shortcomings regarding the dynamic scaffold culture used in the current study, we believe that effects observed in this setting are most predictive for the in vivo situation. 

As expected, pro-inflammatory cytokines used at high concentrations (10 ng/mL for IL-1β and 50 ng/mL for TNF-α) exerted the strongest effects on ASC functional properties. This provides a proof of principle that the inflammatory environment impacts on the parameters investigated in the current settings. However, while corresponding to the experimental design in several previous studies [39,40,41,42], these high cytokine concentrations are artificial and unlikely to be found in vivo. Therefore, we also used the same cytokines at low concentrations (0.01 ng/mL for IL-1β and 0.1 ng/mL for TNF-α). These are closer to the concentrations found in peritendineum and serum of patients with Achilles tendon rupture and chronic Achilles tendon disease, respectively [43,44], although comprehensive data on cytokine concentrations in acute tendon disease are still lacking. Interestingly, at these low concentrations, the only minor differences were observed compared to the controls, but there was a remarkable increase in variability of data between donors for many parameters. While this is already evident considering the high interquartile ranges of data, it even entailed that there were several extreme outlier values, which we had to remove from the boxplot graphics as they strongly compromised their clarity. This was dissatisfactory, but might actually underline that biological variability makes it difficult to predict clinical outcomes in different patients. Yet, it is still possible that results at low cytokine concentrations would have been more consistent after longer observation periods, as for several parameters, there were differences between Day 1 and Day 3, indicating that ASC response to these low-dose stimuli might be slower. Remarkably, co-culture with leukocytes, which we chose as the most natural stimulus, again led to consistent effects, although we had expected the highest variability of data in this condition, due to the increased complexity of the model. Furthermore, we had anticipated to see differences between co-cultures with activated leukocytes compared to non-activated leukocytes, based on the increased production of pro-inflammatory cytokines by peripheral blood leukocytes upon concanavalin A stimulation, which we had investigated beforehand [31]. However, this was not the case; hence, the activation state of leukocytes did not play a major role with respect to the parameters investigated in the current study. This suggests that the effects observed were not only due to the presence of pro-inflammatory cytokines in a dose-dependent manner, but that other soluble factors and cell-cell contacts also played an important role which still has to be elucidated.

In accordance with the current data, IL-1β used at 1 to 5 ng/mL was shown to alter glucose metabolism and inhibit adipogenic and chondrogenic differentiation and to decrease tendon marker expression in injured mouse Achilles tendon progenitor cells [42]. Furthermore, TNF-α alone used at low concentrations (0.0025 ng/mL) inhibited proliferation, as well as tendon- and bone-related marker expression in rat tendon-derived progenitor cells, but increased these parameters when combined with TGF-β1, suggesting synergistic effects [45]. Similar as observed for IL-1β and TNF-α in the current study, IL-6 used at 0.1 to 100 ng/mL increased proliferation but inhibited tenogenic marker expression in rat tendon-derived progenitor cells, with significant effects on scleraxis expression observed at 10 ng/mL or higher [46]. While the current, as well as these previous studies, consistently underline that inflammatory environment compromises tenogenic differentiation, it should be acknowledged that at the moment, the body of evidence is not yet comprehensive and further studies are required to understand the complex interplay of different factors in tenogenic differentiation.

In one of these previous studies focusing on tenogenic differentiation, osteogenic differentiation was also compromised in the presence of IL-1β [42], which stands in contrast to the current study. Potentially, this difference between studies is due to the different cell types investigated, as it was previously reported that an inflammatory environment, represented by TNF-α, impeded osteogenic differentiation in periodontal ligament stem cells, while bone marrow-derived progenitor cells appeared to react contrarily, with significant effects observed already at low cytokine concentrations such as 0.01 ng/mL [47]. This issue warrants further investigation into the osteogenic differentiation of non-tendon or -ligament-derived progenitor cells in inflammatory environment, particularly as erroneous osteogenic differentiation of mesenchymal progenitor cells may lead to unwanted calcifications within tendons. Yet, in the equine in vivo model, non-invasive imaging and histology has so far failed to detect any calcifications in treated tendons [8,48].

Co-culture with leukocytes, irrespective of their activation state, decreased ASC proliferation in contrast to pro-inflammatory cytokines exerted similar, although fewer, effects on musculoskeletal marker expression as did high cytokine concentrations. With respect to the tenogenic potential of ASC in the inflammatory environment, this included the finding that scleraxis was downregulated in presence of leukocytes throughout all culture conditions. It appears possible that although ASC are generally capable of supporting regeneration by a variety of mechanisms, including differentiation as well as immunomodulation, there may be only one predominating mechanism at a time. In the current experimental setting, the active interplay occurring between ASC and peripheral blood leukocytes in direct co-culture [31] possibly prevents ASC differentiation into tissue-specific cells. This supports the assumption that cell replacement by differentiation into the required cell type is not the predominating mechanism of action of mesenchymal progenitor cells. The latter may apply particularly when the cells are transplanted into an inflammatory environment, such as in acute tendon disease, even though for cell-based tendon therapy, ASC survival and engraftment have been demonstrated [19,20].

The current data underline the need for more realistic in vitro models, since not all findings were shared between the standard monolayer culture and (dynamic) scaffold culture or between supplementation with pro-inflammatory cytokines and co-culture with leukocytes. Nevertheless, collectively, this study demonstrates that tenogenic properties of ASC are compromised in inflammatory environment, as this was observed consistently in the different experimental settings. 

## 4. Materials and Methods

### 4.1. ASC Recovery and Characterization 

Adipose tissue was collected aseptically from seven horses (mean age: 4.6 years; age range: 1–12 years) sacrificed for unrelated reasons. As described previously [22], mononuclear cells were isolated by collagenase digestion and seeded in Dulbecco’s Modified Eagle Medium (1 g/L glucose; Gibco^®^, ThermoFisher Scientific, Darmstadt, Germany) supplemented with 10% fetal bovine serum, 1% penicillin-streptomycin and 0.1% gentamycin. Cells were incubated in a humidified atmosphere at 37 °C and 5% CO_2_, passaged upon subconfluency, and then cryopreserved. All experiments were repeated with ASC from each individual donor animal (*n* = 7) at passage 3.

ASC proliferation at standard and inflammatory conditions was assessed based on monolayer cultures seeded at 3000 cells per cm^2^. After three days of pre-incubation, pro-inflammatory cytokines or leukocytes were added as described below. Standardized phase-contrast microscopy images were captured (DMi1, Leica Microsystems, Wetzlar, Germany) at Day 1 and Day 3 of inflammatory stimulation, and subjected to quantitative image analysis to assess confluency as a measure of proliferation. Furthermore, on Day 3, additionally prepared monolayer cultures from two animals were detached, counted and generation times were calculated as previously described [7] to confirm that confluency was representative for cell proliferation.

ASC trilineage differentiation at standard and inflammatory conditions was performed using the STEMPRO^®^ Adipogenesis, Osteogenesis and Chondrogenesis Differentiation Kits (Gibco^®^, ThermoFisher Scientific). For adipogenic and osteogenic differentiation, cells were seeded at 3000 or 2000 cells per cm^2^, respectively, and differentiation medium was added after three days of pre-incubation. Chondrogenic differentiation was performed in pellet cultures, which were obtained by centrifugation of 500,000 ASC in chondrogenic differentiation medium at 280 g for 5 min. Adipogenic differentiation medium was additionally supplemented with 5% rabbit serum (Sigma-Aldrich, Steinheim, Germany), and all differentiation media were supplemented with pro-inflammatory cytokines according to the experimental groups detailed below. Adipogenic differentiation cultures were incubated for four days, while osteogenic and chondrogenic differentiation cultures were incubated for 21 days. Subsequently, adipogenic and osteogenic cultures were stained with Oil Red O or von Kossa, respectively, and paraffin sections of the chondrogenic micromasses were stained with Alcian blue. Standardized images were captured (DMi1, Leica Microsystems) and subjected to quantitative analysis.

### 4.2. Preparation and Seeding of Tendon Scaffolds

Equine distal limbs were collected at an abattoir, and healthy superficial digital flexor tendons were excised from the metacarpal regions aseptically. Tendons were then subjected to decellularization by combining freeze-thaw cycles and treatment with Triton X-100 (Carl Roth GmbH & Co. KG, Karlsruhe, Germany) as described and evaluated previously [49,50]. 300 µm-thick tendon extracellular matrix scaffolds were then prepared by sectioning the tendons longitudinally using a cryostat (CM 3050 S, Leica Biosystems, Wetzlar, Germany). Scaffolds intended for mechanical stimulation in the bioreactor were sectioned to 7 cm in length and 1 cm in width, while scaffolds intended for static culture were 1 cm in length and width.

The scaffolds were seeded by evenly distributing 30 µL of cell culture medium containing 300,000 cells per cm^2^ on the scaffold surface. ASC were allowed to attach for 6 h, then scaffolds were covered with cell culture medium and pre-incubated for three days before the stimulation experiments were started. 

Monolayers cultures were seeded at a density of 3000 cells per cm^2^ at the same time point. 

### 4.3. Dynamic Culture of Tendon Constructs in the Cyclic Strain Bioreactor

For tenogenic induction by physiological stimuli, namely the extracellular tendon matrix and cyclic stretching, ASC-seeded tendon scaffolds were placed in a custom-made cyclic strain bioreactor. This bioreactor, which was manufactured at the Institute of Technical Chemistry, Leibniz University, Hanover, Germany, allows to fix three technical replicate constructs in its medium chamber and to apply displacement-controlled cyclic strain at variable parameters via a 1 kN motor and an integrated software. For Illustration, see Figure S4. The daily applied mechanical stimulation regime consisted of 15 min cyclic stretching, followed by 15 min relaxation and another period of 30 min cyclic stretching (1 Hz, 2% strain, without pre-loading). 

In parallel to these dynamic culture experiments, static scaffold and monolayer cultures were maintained without any mechanical stimulation or static strain, and subjected to inflammatory culture conditions according to the same schedule.

### 4.4. Leukocyte Recovery and Inflammatory Culture Conditions

Heparinized peripheral blood was collected from a healthy donor horse (gelding, 4 years) by puncturing the jugular vein, as approved by the local ethics committee (Landesdirektion Leipzig, N08/17, March 2017). Peripheral blood leukocytes were isolated by density gradient centrifugation of 1:2 diluted whole blood on Leuko Spin Medium (1.090 g/mL, pluriSelect Life Science, Leipzig, Germany) at 800× *g* for 20 min without brakes. The leukocyte layer was then carefully collected and washed with washing buffer (phosphate buffered saline with 0.5% bovine serum albumin (Sigma-Aldrich) and 2 mM ethylenediaminetetraacetic acid (Carl Roth GmbH & Co. KG)) [31]. Numbers of viable leukocytes were determined using Trypan blue and a hemocytometer. Part of the obtained leukocytes were then subjected to stimulation with concanavalin A (2.5 µg/mL, Sigma-Aldrich) for 1 h, before being used in the respective activated leukocyte co-culture groups.

Different inflammatory conditions were then established to evaluate their impact on ASC proliferation, trilineage differentiation, viability, tenogenic differentiation, and tendon scaffold repopulation. These included either the direct co-culture of ASC with allogeneic, non-activated, or activated leukocytes in a ratio of 1:10, or the supplementation of culture medium with pro-inflammatory cytokines. For the latter, IL-1β and TNF-α were used alone or in combination, both either at low concentrations (IL-1β-low: 0.01 ng/mL; TNF-α-low: 0.1 ng/mL) or at high concentrations (IL-1β-high: 10 ng/mL; TNF-α-high: 50 ng/mL). 

All different inflammatory conditions were tested in the monolayer cultures. In static scaffold cultures, all conditions involving the pro-inflammatory cytokines, as well as the co-culture with non-activated leukocytes were applied, whereas in dynamic scaffold cultures, only the pro-inflammatory cytokine combinations and the co-culture with non-activated leukocytes were used (Table 1). For trilineage differentiation, all conditions involving pro-inflammatory cytokines but no co-culture with leukocytes were included. 

Leukocytes or cytokines were added to the ASC cultures after three days of pre-incubation and before cyclic stretching was applied in the dynamic culture group (day 0). Unless stated otherwise, samples were then harvested and washed in phosphate buffered saline, with the leukocytes in the supernatants being discarded, and analyzed at Day 1 and Day 3. 

### 4.5. Viability Staining and Histology

For viability staining, monolayer and scaffold cultures were stained using the LIVE/DEAD™ Viability/Cytotoxicity Kit (Invitrogen™, ThermoFisher Scientific) according to the manufacturer’s instructions. With regard to the dynamic scaffold cultures, the technical replicates of the staining were prepared from two different regions (central and end regions, but not including the clamped areas) of the stretched scaffolds. Directly after staining, three images were obtained from each replicate at standardized settings using a Keyence HS All-In-One Fluorescence Microscope (Keyence, Osaka, Japan). Viable cell morphology and distribution were evaluated qualitatively, and the count of dead cells was obtained by quantitative image analysis.

For histology, ASC-seeded scaffold samples, again from two different regions for stretched constructs, were fixed in 4% paraformaldehyde and embedded in paraffin. Sagittal 5 µm sections were then prepared and stained with hematoxylin and eosin. Two sections from each sample were evaluated and scored microscopically (DMi1, Leica Biosystems, 10× objective) by two independent and blinded observers, as detailed in Table 2, focusing on the efficiency of scaffold repopulation by the ASC.

### 4.6. Image Analysis

All computations were performed using Mathematica version 11.3 (Wolfram Research, Inc., Champaign, IL, USA). The implementation of the color deconvolution algorithm described below has been made freely available [51]. Additionally, several other free open-source applications exist, including a plugin for ImageJ [52]. In the following description of the methods used, image pixel values were assumed to be in the range of [0,1].

#### 4.6.1. Background Illumination Correction

The quantification of the adipogenic, chondrogenic, and osteogenic differentiation images required constant illumination and coloring. Therefore, these images were captured following a rigorous protocol to ensure consistent imaging results that allowed for automated analysis. Thus, before each session of acquisition, the operator adjusted the brightness of the microscope to a previously determined, optimal level. As a next step, settings of the camera such as gain, exposure, etc. were set to a default value, and the automatic adjustment for the camera was turned off.

To correct the illumination of the images, a brightfield reference was obtained by capturing an empty slide of the same type that was used for the histological samples. Similarly, a darkfield reference was acquired by closing the light-way to the camera to correct for erroneous pixels of the camera sensor. Using these two references, the illumination of all acquired images were corrected by calculating:

CorrectedImage = (OriginalImage − Darkfield)/(Brightfield − Darkfield)(1)

#### 4.6.2. Color Deconvolution

One significant step in quantifying the Oil Red O and Alcian blue stainings was to separate each image into respective concentration images for each dye. With the Color Deconvolution algorithm [53], each pixel is regarded as a mixture of different dyes with unknown concentration. By empirically estimating a set of color vectors for particular stainings (so-called deconvolution kernels), the method can then untangle the image colors and calculate the intensity images that represent the concentrations of each dye.

A custom tool for estimating correct color vectors was implemented, that allowed the dye colors to be adjusted, and the deconvolution results to be inspected dynamically. This tool is based on previous work [54] that suggested the use of polar coordinates for the color vectors. The final deconvolution kernel for both the Oil Red O and the Alcian blue staining was found by assembling an image collage from nine randomly selected images, and by adjusting for optimal color vector settings regarding the observed concentration images and pixel intensity statistics. These two deconvolution kernels were used to process all brightness-corrected Oil Red O and Alcian Blue images.

#### 4.6.3. Analysis of Adipogenic Differentiation

In a first step, a cell mask was computed on the luminance channel using a local-adaptive binarization algorithm, which takes a region around each pixel, calculates the local mean and standard deviation, and applies thresholding based on these values and the pixel value itself. As this is computationally expensive, images were down-scaled to a width of 512 pixels. The size of the local region depends on the microscope magnification. For images acquired with a magnification of 10× and 20×, a local region of 30 and 60 pixels was used, respectively.

Since cells were darker than the bright background, the negated luminance image was used for binarization and all pixel-values above 1.1×mu−0.3×sigma, where mu and sigma is the local mean and standard deviation, were replaced with 1 and all others with 0. Images were assumed to have a pixel-value in the interval [0,1]. After upscaling the mask to its original dimensions, all analysis was restricted to the regions of the cells.

In a second step, the color deconvolution using the estimated Oil Red O kernel was computed. With the cell mask and the dye concentration images, all statistically analyzed cell areas and concentration percentages were computed.

#### 4.6.4. Analysis of Chondrogenic Differentiation

Similar to the approach in Section 4.6.2, the color deconvolution using the Alcian blue deconvolution kernel was computed, and analysis was restricted to the area of the pellets. However, for the mask of the pellets, a binarization using an automatically determined, global threshold was sufficient. The computation of the threshold was done using the Otsu cluster variance maximization method [55]. For the binarization, the luminance channel was downscaled to a third of the original image width (864 pixels). After thresholding, a morphological closing with a radius of 4 was employed to close smaller holes in the binarized pellet. Furthermore, all but the largest object in the binary mask were deleted to remove artifacts such as air-bubbles. The remainder of this analysis was similar to the method used for the adipogenic differentiation.

#### 4.6.5. Analysis of Osteogenic Differentiation

For the single dye von Kossa stainings, the luminance channel of the brightness-corrected images was used directly. This approach was justified because the staining itself does not contain a mixture of prominent colors, and darker regions already indicate stronger differentiation. Therefore, the mean pixel intensity was calculated by dividing the total of all pixel values by the size of the image. The obtained value was subtracted from 1 to get a measure that is larger for darker images.

#### 4.6.6. Analysis of Unstained Phase Contrast Images

For the phase contrast images, a global binarization threshold was calculated for each separate image to obtain the cell area. Empirical tests showed that adding a value of 0.07 to the mean intensity of the image provided a consistent threshold that was used for all images. The result of this analysis was the percentage of the mask area within the image.

#### 4.6.7. Analysis of LIVE/DEAD

For this quantification, the same approach as presented in [36] was employed (also see Figure S5). However, due to the high variance in the morphology of the viable cells, the method was only used for quantifying dead cells.

### 4.7. Real-Time RT-PCR

Frozen ASC-seeded tendon scaffolds were homogenized using a Tissue Lyser II (Qiagen, Hilden, Germany) and samples were treated with proteinase k (Qiagen) at 55 °C. Total RNA of scaffold-seeded and monolayer ASC was then isolated using the RNeasy^®^ Mini Kit with On Column DNase digestion (Qiagen). RNA was converted to first strand complementary DNA (cDNA) using Reverse Transcriptase RevertAid H Minus (ThermoFisher Scientific). Next, cDNA was mixed with the respective primers and iQ™ SYBR Green Supermix (Bio-Rad Laboratories, Munich, Germany). Primer sequences for target and housekeeping genes are given in Table 3. Relative quantification of cDNA was performed using an Applied Biosystems™ 7500 Real Time PCR System, and gene expression ratios were calculated according to [56]. Additionally, for further comparisons between groups as well as for graphical presentation of data, and gene expression was normalized to the corresponding controls within each group (monolayer, static, and dynamic scaffold cultures without inflammatory stimulation).

### 4.8. Statistical Analysis

Statistical analysis was performed using SPSS Statistics 23 software (IBM Deutschland GmbH, Ehningen, Germany). As data were not normally distributed, non-parametric Friedman and Wilcoxon post-hoc tests were used. Extreme outlier values remained included during these analyses but were excluded from the boxplot graphics to improve their clarity. Differences were considered significant at *p* < 0.05. 

## Figures and Tables

**Table 1:** Overview of the experimental groups.

Culture Condition	Treatment Groups
Monolayer	Control
	IL-1β-high
	TNF-α-high
	IL-1β-low
	TNF-α-low
	IL-1β-high + TNF-α-high
	IL-1β-low + TNF-α-low
	Leuko-stim
	Leuko
Static scaffold culture	Control
	IL-1β-high
	TNF-α-high
	IL-1β-low
	TNF-α-low
	IL-1β-high + TNF-α-high
	IL-1β-low + TNF-α-low
	Leuko
Dynamic scaffold culture	Control
	IL-1β-high + TNF-α-high
	IL-1β-low + TNF-α-low
	Leuko

IL-1β-high: 10 ng/mL; IL-1β-low: 0.01 ng/mL; TNF-α-high: 50 ng/mL; TNF-α-low: 0.1 ng/mL; Leuko-stim: co-culture with activated leukocytes; Leuko: co-culture with non-activated leukocytes.

**Table 2:** Score system used for blinded evaluation of histological sections.

Category	Description	Score Points
A. Cell distribution at seeded scaffold surface	No cells	0
	Single cells	1
	Monolayer	2
	Partial multilayer	3
	Constant multilayer	4
B. Cell integration within the scaffold	No cells integrated	0
	<10% of cells integrated	1
	10–50% of cells integrated	2
	> 50% of cells integrated	3
C. Cell distribution at opposite scaffold surface	No cells	0
	<10% of cells at opposite side	1
	10–50% of cells at opposite side	2
	>50% of cells at opposite side	3

**Table 3:** Primers used for real-time RT-PCR.

Gene	Primer Sequences	Accession Number	PCR Product (bp)
*ACTB*	For: ATCCACGAAACTACCTTCAAC	NM_001081838.1	174
	Rev: CGCAATGATCTTGATCTTCATC		
*GAPDH*	For: TGGAGAAAGCTGCCAAATACG	NM_001163856.1	309
	Rev: GGCCTTTCTCCTTCTCTTGC		
Collagen 1A2	For: CAACCGGAGATAGAGGACCA	XM_001492939.3	243
	Rev: CAGGTCCTTGGAAACCTTGA		
Collagen 3A1	For: AGGGGACCTGGTTACTGCTT	XM_001917620.3	216
	Rev: TCTCTGGGTTGGGACAGTCT		
Decorin	For: ACCCACTGAAGAGCTCAGGA	NM_001081925.2	239
	Rev: GCCATTGTCAACAGCAGAGA		
Tenascin-C	For: TCACATCCAGGTGCTTATTCC	XM_001916622.3	163
	Rev: CTAGAGTGTCTCACTATCAGG		
Osteopontin	For: TGAAGACCAGTATCCTGATGC	XM_001496152.3	158
	Rev: GCTGACTTGTTTCCTGACTG		
Scleraxis	For: TACCTGGGTTTTCTTCTGGTCACT	NM_001105150.1	51
	Rev: TATCAAAGACACAAGATGCCAGC		
Mohawk	For: AAGATACTCTTGGCGCTCGG	XM_014737017.1	170
	Rev: ACACTAAGCCGCTCAGCATT		
Smad8	For: AGCCTCCGTGCTCTGCATT	AB106117.1	200
	Rev: CCCAACTCGGTTGTTTAGTTCAT		

## Supplementary Materials

Supplementary materials can be found at http://www.mdpi.com/1422-0067/19/9/2549/s1.

## Abbreviations

ASC	Adipose-derived stromal cells
FC	Fold change
IL	Interleukin
Leuko	Leukocytes
Leuko-stim	Leukocytes stimulated with concanavalin A
MSC	Multipotent mesenchymal stromal cells
RT-PCR	Reverse transcription polymerase chain reaction
TGF	Transforming growth factor
TNF	Tumor necrosis factor
