# Supplementary material for: Tenogenic Properties of Mesenchymal Progenitor Cells Are Compromised in an Inflammatory Environment
Source: Int J Mol Sci. 2018 Aug 28;19(9):2549. doi: 10.3390/ijms19092549 (PMC6163784; doi:10.3390/ijms19092549)

Table S1. Generation times of adipose-derived stromal cells (ASC) in different inflammatory environments.

Inflammatory Condition	Generation Time (days)	
	Donor 1	Donor 2
IL-1β-high (10 ng/mL)	1.52	1.38
IL-1β-low (0.01 ng/mL)	1.89	2.33
TNF-α-high (50 ng/mL)	1.58	1.44
TNF-α-low (0.1 ng/mL)	1.79	2.27
IL-1β-high + TNF-α-high	1.83	2.00
IL-1β-low + TNF-α-low	1.85	2.00
control	2.42	2.65

Figure S1. Musculoskeletal gene expression in absence of inflammatory stimulation. The boxplots show the relative target gene expression of adipose-derived stromal cells (ASC) in monolayer culture, static scaffold culture or dynamic scaffold culture, in absence of inflammatory stimulation. Groups displayed by boxes sharing the same letter are significantly different from each other; hash marks (#) indicate significant differences between day 1 and day 3 ($p < 0.05$; $n = 7$).

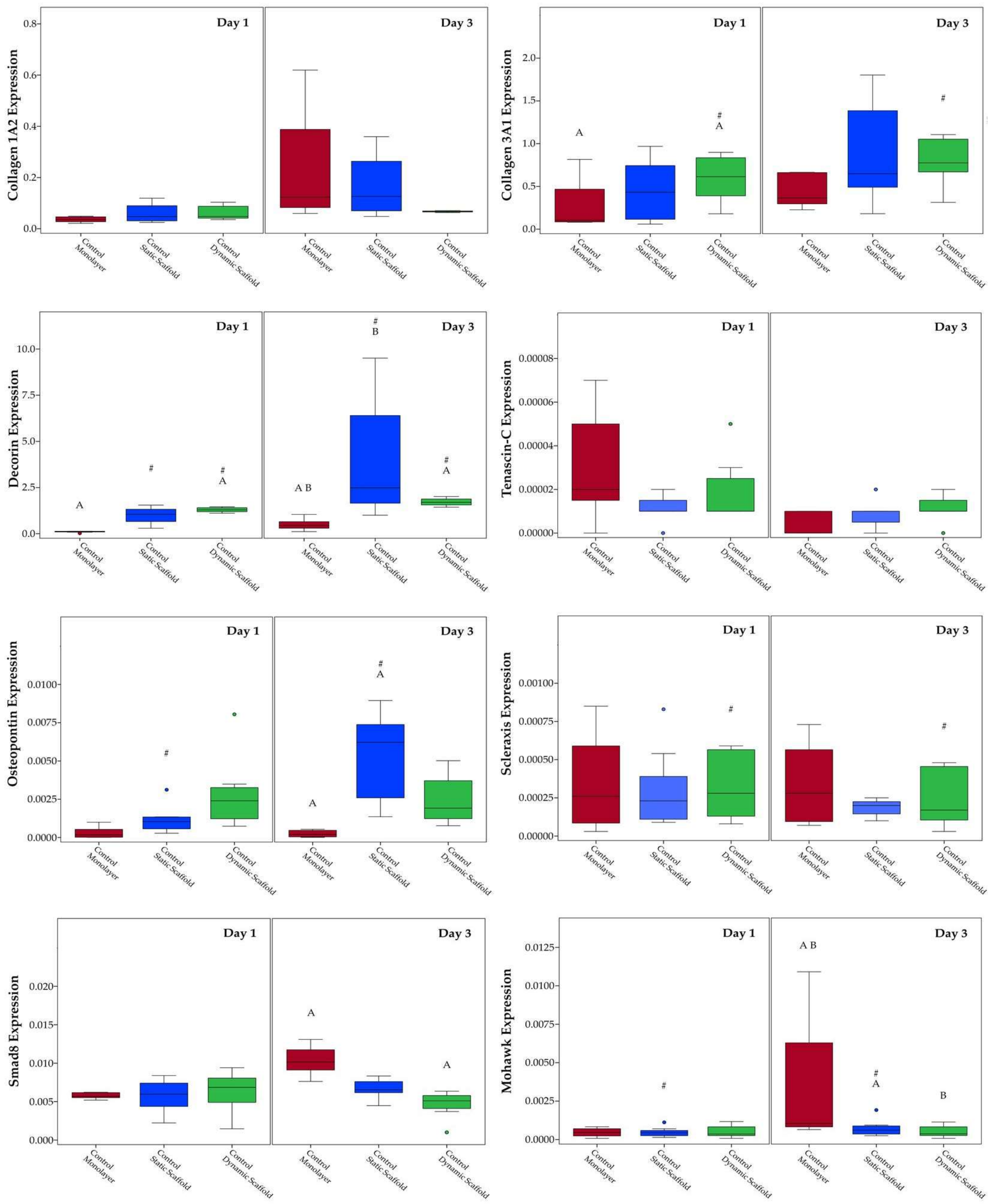

Figure S2. Tendon-specific gene expression patterns. The bar graphs illustrate the expression pattern of collagen 1A2, scleraxis and tenascin-C in adipose-derived stromal cell (ASC) monolayer, static and dynamic scaffold culture without inflammatory stimulation (controls; upper row) and in dynamic scaffold culture in different inflammatory environments (IL-1β-high: 10 ng/mL; IL-1β-low: 0.01 ng/mL; TNF-α-high: 50 ng/mL; TNF-α-low: 0.1 ng/mL; Leuko: co-culture with non-activated leukocytes; second row). Note that an expression pattern corresponding to healthy tendon tissue, with high collagen 1A2, medium scleraxis and low tenascin-C expression [33], is only maintained in scaffold culture with no or mild inflammatory stimulation. Bars show the median values, error bars the 50% confidence interval. The latter was chosen since standard 95% confidence intervals could not be displayed for some groups that contained zero values. The boxplots (lower row) show the ratio of collagen 1A2 to collagen 3A1 expression in ASC monolayer, static and dynamic scaffold culture without inflammatory stimulation (controls; upper row) and in dynamic scaffold culture in different inflammatory environments (IL-1β-high: 10 ng/mL; IL-1β-low: 0.01 ng/mL; TNF-α-high: 50 ng/mL; TNF-α-low: 0.1 ng/mL; Leuko: co-culture with non-activated leukocytes; second row). While this ratio is high in healthy tendon tissue, this was not reflected in dynamic scaffold culture and further compromised in inflammatory environment. However, differences were not significant.

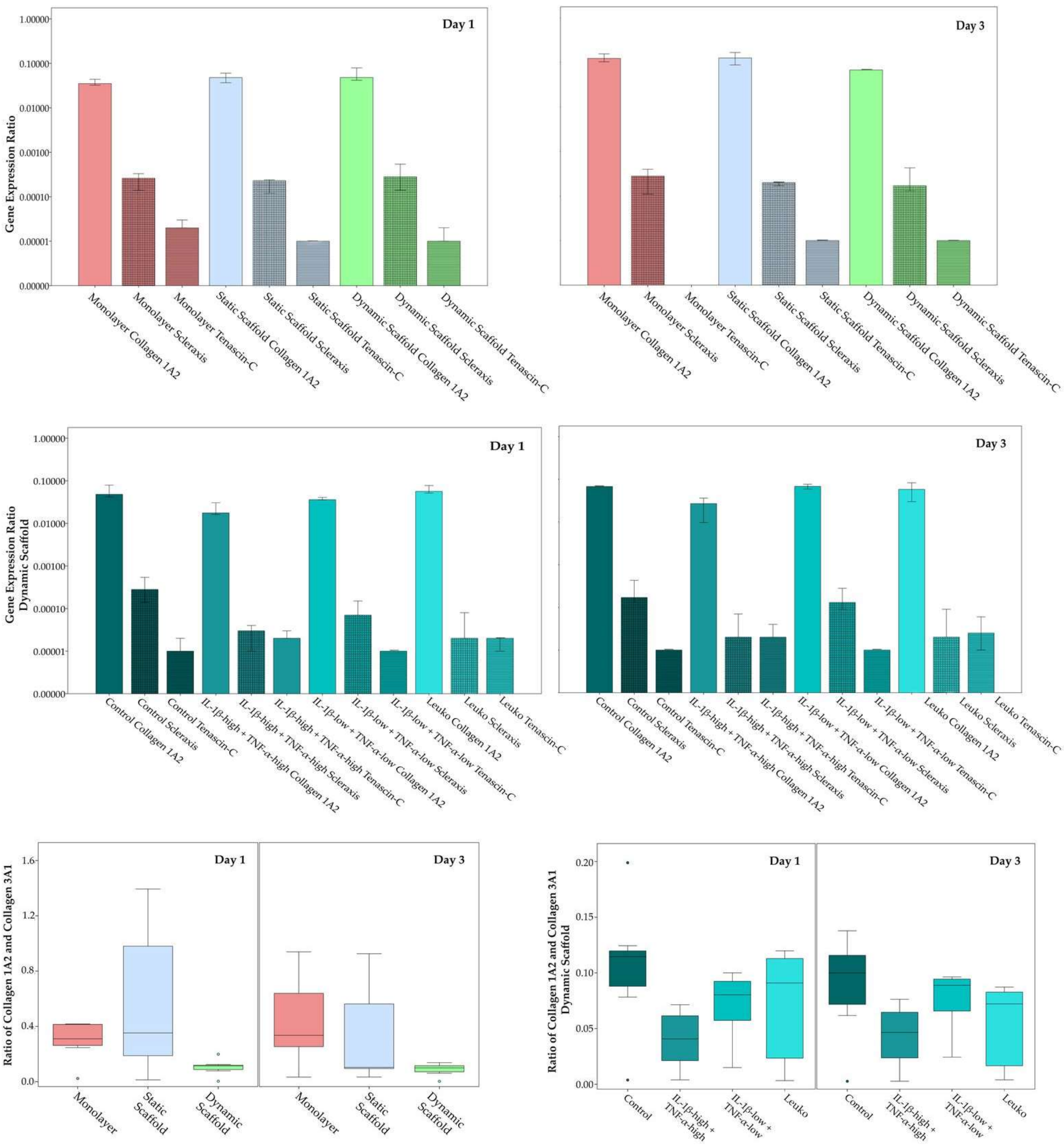

Figure S3. Scoring of histological sections. Boxplots display the score points obtained within different categories when evaluating scaffold repopulation efficiency in static and dynamic conditions as well as in different inflammatory environments (IL-1β-high: 10 ng/mL; IL-1β-low: 0.01 ng/mL; TNF-α-high: 50 ng/mL; TNF-α-low: 0.1 ng/mL; Leuko: co-culture with non-activated leukocytes). Total score points are shown in the main manuscript. Groups displayed by boxes sharing the same letter or symbol are significantly different from each other; hash marks (#) indicate significant differences between day 1 and day 3 ($p < 0.05$).

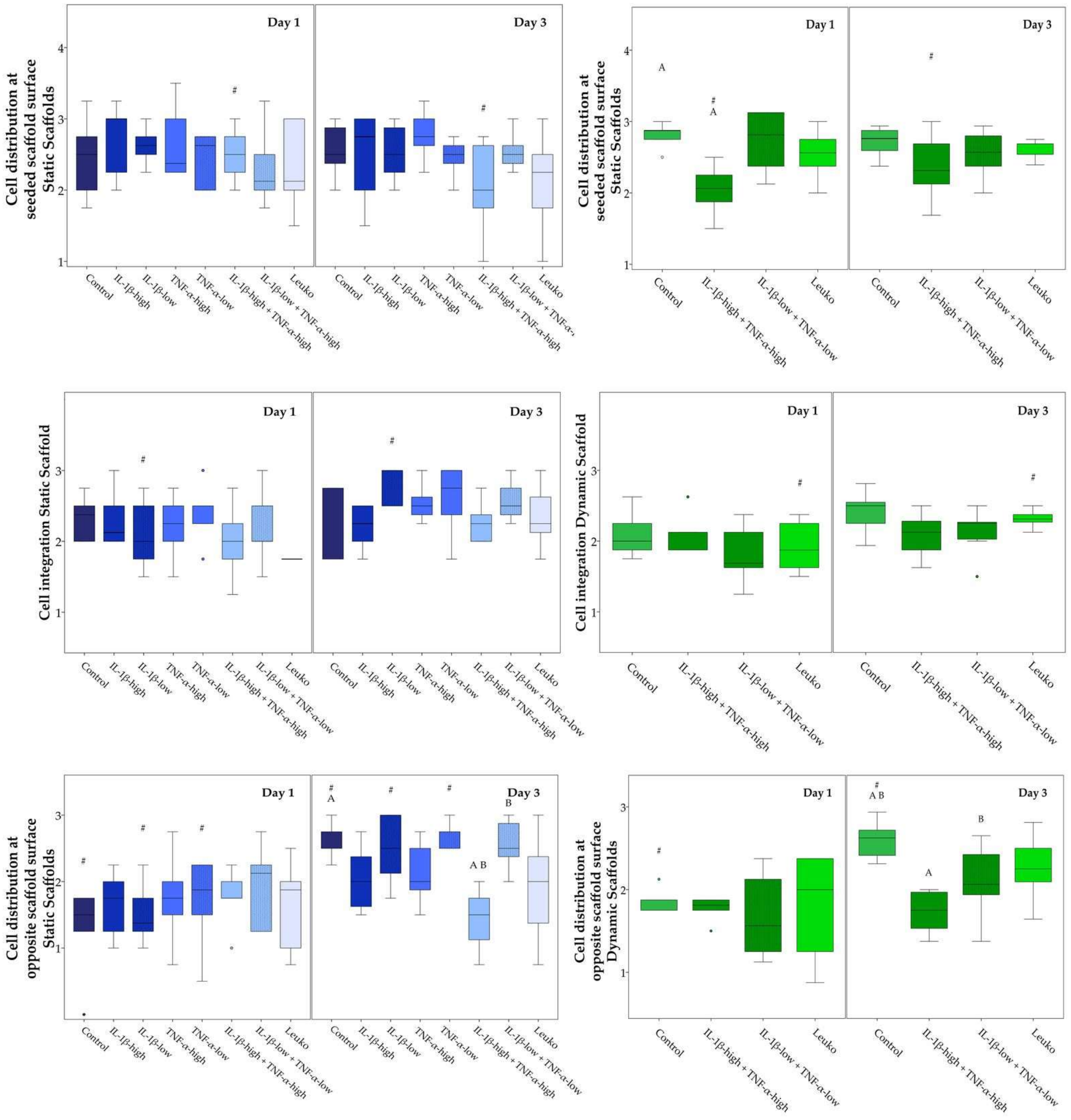

Figure S4. Cyclic strain bioreactor. Custom-made cyclic strain bioreactor used for stretching of cell-seeded tendon scaffolds. 1: motor unit; 2: medium chamber with space to fix three tendon constructs; 3: connection and drive shaft.

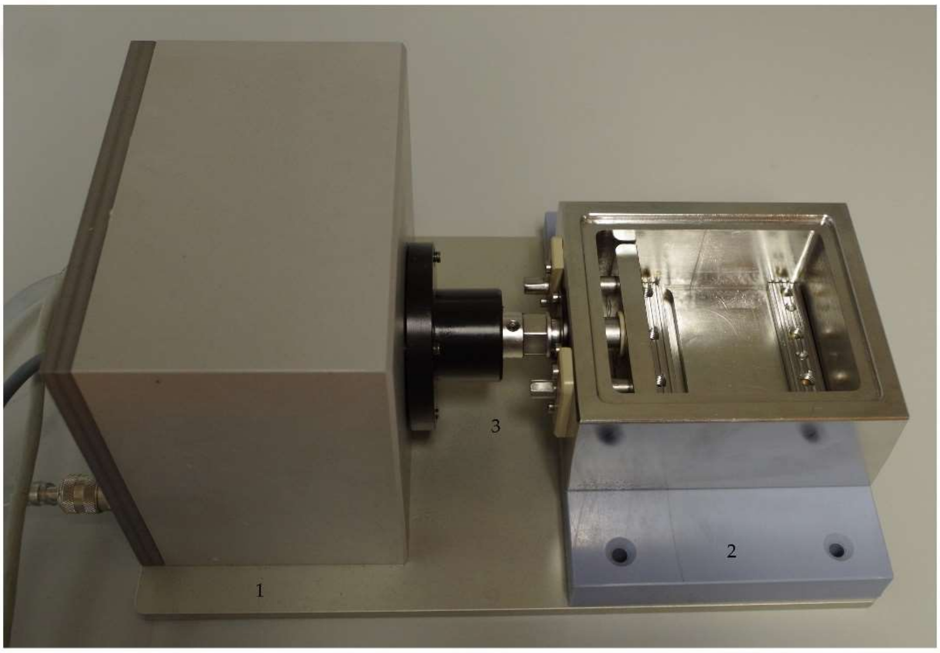

Figure S5. LIFE/DEAD™ staining and segmentation of dead cells. Exemplary images of control adipose-derived stromal cells (ASC) in monolayer culture (first row (a)), static scaffold culture (second row (b)) and dynamic scaffold culture (third row (c)) without inflammatory stimulation after LIVE/DEAD™ staining at day 3; the left column (a1 to c1) displays overlay images, with viable cells shown in green and dead cells in red; the right column (a2 to c2) illustrates the segmentation of dead cells, with white borders marking the dead cells.

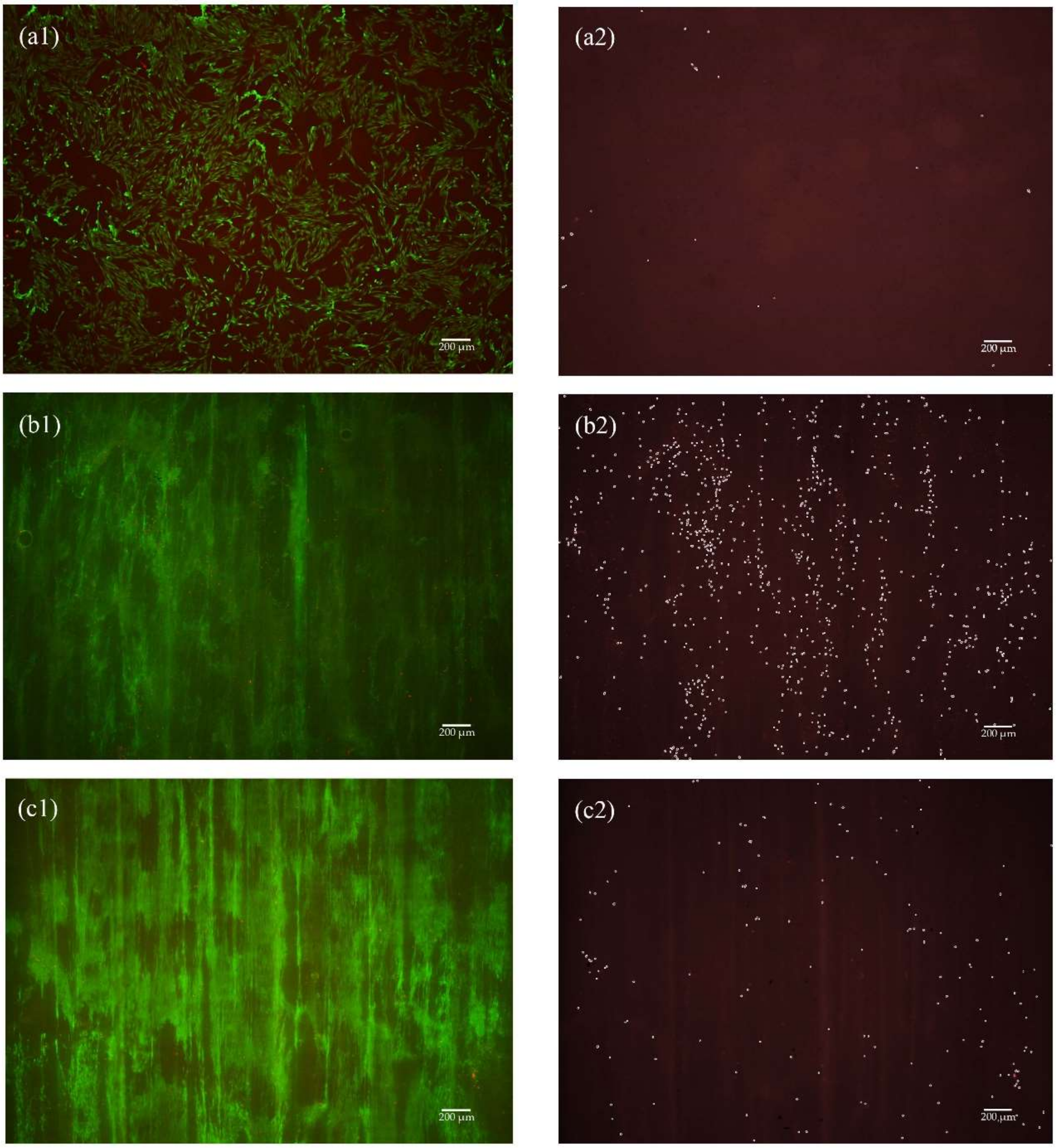

Supplement: Supplementary file 1 [file ijms-19-02549-s001.pdf]
